# Spinal Cord Infarction: Clinical and Neuroradiological Clues of a Rare Stroke Subtype

**DOI:** 10.3390/jcm14041293

**Published:** 2025-02-15

**Authors:** Marialuisa Zedde, Arturo De Falco, Carla Zanferrari, Maria Guarino, Francesca Romana Pezzella, Shalom Haggiag, Gianni Cossu, Rocco Quatrale, Giuseppe Micieli, Massimo Del Sette, Rosario Pascarella

**Affiliations:** 1 Neurology Unit, Stroke Unit, Azienda Unità Sanitaria Locale-IRCCS di Reggio Emilia, Viale Risorgimento 80, 42123 Reggio Emilia, Italy; 2 Neurology and Stroke Unit, Ospedale del Mare, ASL Napoli 1 Centro, 80147 Naples, Italy; arturodefalco@tin.it; 3 Neurology and Stroke Unit, ASST Melegnano-Martesana, 20070 Milan, Italy; carla.zanferrari@asst-melegnano-martesana.it; 4 IRCCS Istituto Delle Scienze Neurologiche Di Bologna, 40139 Bologna, Italy; maria.guarino@aosp.bo.it; 5 Stroke Unit, Dipartimento Di Neuroscienze, Azienda Ospedaliera San Camillo Forlanini, 00152 Rome, Italy; frpezzella@gmail.com; 6 Neurology Unit, Dipartimento Di Neuroscienze, Azienda Ospedaliera San Camillo Forlanini, 00152 Rome, Italy; neuroshalom@hotmail.com; 7 Neurology Unit, Department of Neuroscience, ARNAS Brotzu, 09047 Cagliari, Italy; giovannicossu1@gmail.com; 8 Dipartimento Di Scienze Neurologiche, UOC di Neurologia—Ospedale dell’Angelo—ULSS 3 Serenissima, 30174 Venezia-Mestre, Italy; rocco.quatrale@aulss3.veneto.it; 9 Former Department of Emergency Neurology, IRCCS C. Mondino Foundation, 27100 Pavia, Italy; giuseppe.micieli53@gmail.com; 10 Neurology Unit, IRCCS Ospedale Policlinico San Martino, 16132 Genoa, Italy; massimo.delsette@hsanmartino.it; 11 Neuroradiology Unit, Azienda Unità Sanitaria Locale-IRCCS di Reggio Emilia, Viale Risorgimento 80, 42123 Reggio Emilia, Italy; pascarella.rosario@ausl.re.it; 12 Associazione Neurologia di Emergenza Urgenza (ANEU), 53100 Siena, Italy

**Keywords:** spinal cord infarction, time, MRI, owl’s eyes, DWI, periprocedural, dissection, aorta

## Abstract

Spinal cord infarction (SCI) of arterial origin is a rare vascular event, and its incidence is probably underestimated. There are no strong epidemiological data, and the diagnostic pathway is complex and sometimes incomplete. Furthermore, many cases may be misdiagnosed as other forms of acute and subacute myelopathies. The focus of this review is the clinical and neuroradiological issues in diagnosing SCI and their respective reliability in a clinical setting. The new proposed diagnostic criteria of SCI, although not covering all aspects, highlight the need for a comprehensive approach, including even atypical cases, as the lack of cord compression on Magnetic Resonance Imaging (MRI) is the only mandatory feature for diagnosis. Some MRI features are supportive of the diagnosis, particularly when the anterior spinal artery territory is involved and diffusion-weighted imaging (DWI) is used. Several etiologies can be considered, considering traditional vascular risk factors and diseases affecting the aorta and its main branches, yet a significant proportion of cases remain without a definite etiology. The strongest predictor of SCI diagnosis is a clinical variable, i.e., a time to nadir of severe deficits < 12 h.

## 1. Introduction

Stroke is a common cerebrovascular disorder, being the second leading cause of death in the world (11.6%) and the third leading cause of death and disability combined [1]. Ischemic stroke accounted for 62.4% of all incident strokes in 2019 (7.63 million), while intraparenchymal cerebral hemorrhage accounted for 27.9% and subarachnoid hemorrhage for 9.7% [2]. However, the vast majority of available epidemiological studies consider stroke as an event limited to a part of the central nervous system (CNS), namely the brain. The possibility of vascular involvement of the spinal cord is not even considered and recorded. In fact, the 1970 World Health Organization (WHO) definition of stroke refers only to cerebral dysfunction and not to the possibility of spinal cord involvement [3]. Even in the latest version of the International Classification of Diseases (ICD11), the definition of stroke does not include the spinal cord, being derived from the WHO definition [4]. The spinal cord is only included in the proposed new definition of cerebrovascular diseases published in 2013, together with the brain and retina as potential sites of neurological dysfunction due to ischemic stroke [5]. Curiously, both the historical (time-based, i.e., a duration of symptoms < 24 h), and the modern (tissue-based, i.e., the lack of definite tissue damage independent from the duration of symptoms) definitions of transient ischemic attack (TIA) include as sites of sudden dysfunction the brain, retina, and spinal cord [6,7,8,9]. In general, vascular diseases of the spinal cord are uncommon yet critical conditions, often presenting with sudden symptom onset, resulting in disability and impacting quality of life [10]. Vascular myelopathies refer to distinct clinical and neuroradiological findings due to any abnormalities of physiological spinal vascularization, depending on either impaired arterial blood supply or venous drainage, leading to spinal cord injury. The clinical presentation of vascular myelopathies is variable, and a considerable clinical overlap with other etiologies of myelopathy still exists. A proper neuroradiological examination is essential for correct diagnosis and treatment.

Thus, it is clear that spinal cord infarctions (SCIs) have been neglected for a long time, and this has certainly influenced the limited knowledge and attention that we have towards this subtype of stroke. SCI has lower prevalence than brain and retina infarction, but it often results in severe disabilities and functional impairment. The aim of this review is to draw attention to arterial SCI and to detail its main diagnostic aspects, both clinical and neuroradiological, in a pragmatical view, as well as highlight the gray areas that require further effort to draw information and guide clinical choices.

## 2. Epidemiological Notes

The incidence of SCI is not well documented, but it is estimated to account for about 1–2% of all strokes [10]. Based on stroke rates (which range from 540,000 to 780,000 cases annually), SCI is estimated to occur in 5000 to 8000 cases each year in the USA [11]. However, this number may be underestimated, as it likely excludes cases associated with major surgeries, which are a leading cause of SCI. Globally, SCI is estimated to occur in 5–8% of all acute myelopathies [12,13]. SCI is identified in about 8% of patients with multilevel aortic disease [14], and the risk significantly increases in those undergoing thoraco-abdominal aortic surgery, where prevalence may reach 33% [15]. A study on 15,304 patients with a ruptured aortic aneurysm or dissection and 74,555 with unruptured aneurysms found that SCI rates were 1/130 for emergency aortic repairs and 1/600 for non-emergency repairs [16]. In a retrospective analysis of 164 patients with acute SCI, nine cases (5.5%) were associated with fibrocartilaginous embolism (FCE). Given the rarity of SCI, prevalence studies are limited [17].

However, it is hard to retrieve reliable information on the incidence from available series. In a population-based study from Stearns and Benton Counties (Minnesota) [18], the age- and sex-adjusted incidence of SCI was 3.1/100,000 person-years [95% confidence interval (CI) 1.6–7.2]. The 95% CI suggested that the true value of incidence based on the precision of the estimate may range between 1.6 and 7.2/100,000 persons. The only estimate for comparison is the incidence of SCI associated with aortic repair, which was approximately 76 patients every year in the USA [19]. The age- and sex-adjusted incidences of primary and secondary SCI were 1.5/100,000 [95% CI 0.6–3.6] and 1.6/100,000 [95% CI 0.6–3.6] person-years, respectively. The age-adjusted incidences among men and women were 1.5/100,000 [95% CI 0.6–3.7] and 4.6/100,000 [95% CI 2.2–8.7] person-years, respectively.

In a recent systematic review and meta-analysis of spontaneous SCI [20], 48% of patients were male and the pooled mean age was 58.7 ± 3.96; in another meta-analysis, the mean age of onset was around 64 years [21]. The age of onset for SCI ranges widely, from early childhood to late adulthood, but the median age is typically in the sixth to seventh decade of life. In fact, SCI primarily affects adults, and is often linked to complications of atherosclerotic vascular disease. Nonetheless, children, including neonates, may also experience spinal cord infarction under certain conditions [22].

Arterial hypertension was reported in 40% of patients as the most common risk factor, followed by smoking (30%), dyslipidemia (29%), and diabetes (16%), while 28% had no reported vascular risk factors [20]. Most cases (65%) occur at the thoracolumbar level, while others affect the mid-cervical region. Cervical-level infarctions are associated with a more severe presentation, often involving autonomic dysfunction and upper extremity impairment.

However, epidemiological data on SCI are affected by a low awareness of SCI compared with other causes of spinal cord injury, meaning the prevalence is probably underestimated. In addition, these data refer mostly to high-income countries, and there are no reliable data about the prevalence and the features of SCI in low- and middle-income countries.

## 3. Arterial Supply to the Spinal Cord

From 1882 to 1886, Albert Wojciech Adamkiewicz published his greatest works concerning the anatomy of the vasculature of the spinal cord [23]. The main issues focused on the significant variability in spinal cord blood supply and on the remarkable spinal anastomotic network. In fact, the spinal cord has one of the most complex blood supplies of any part of the body, and it is necessary to understand some of its complexity before turning to the problem of its vascular disorders [24].

**Figure 1:**
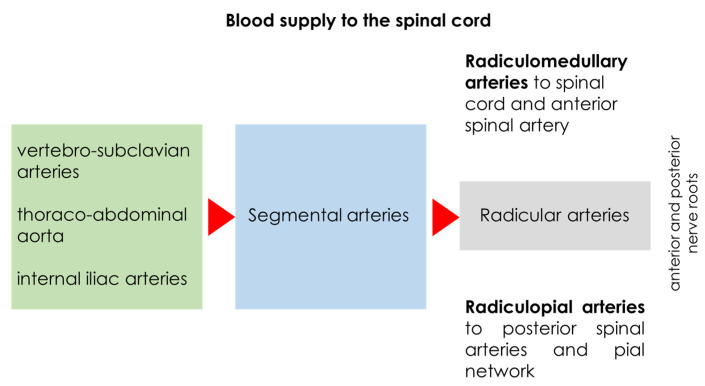
This is a schematic drawing of the main supply of the spinal cord, from the aorta and its branches to the radicular arteries.

The spine’s blood supply consists of paired intersegmental and intercostal arteries that branch from the thoracic aorta. These arteries, named according to the ribs beneath which they travel, run along the posterior sides of each vertebral body. They give off short branches to the vertebral body before dividing into ventral and dorsal branches (as shown in Figure 1). In some cases, an anterior intersegmental connection appears before this division, where the ventral or lateral branch continues as either an intercostal or lumbar artery, depending on the spinal level.

The anterior spinal artery (ASA) originates from two branches of the medial intracranial vertebral arteries, joining at the cervico-medullary junction. This artery runs within the anterior median fissure, supplying the anterior two-thirds of the spinal cord, including the anterior horn cells, corticospinal tracts, and spinothalamic tracts. It has numerous anastomoses along its length and receives radiculomedullary feeders from the vertebral and subclavian arteries, particularly from the artery of cervical enlargement (artery of Lazorthes) around C5 or C6. Angiographically, the ASA typically originates from a common trunk formed by paired vertebral arteries (VAs), located 5–17 mm from the vertebrobasilar junction and often appearing with a distinctive midline hairpin shape. The ASA’s origin varies considerably, with a predominance of one branch over the other or sometimes a unilateral ramus. Rather than a single artery, the ASA is a series of anastomotic loops, continuous in the cervical but less so in the thoracic region. In the cervical region, the ASA receives segmental radiculomedullary arteries from the VA (C1–C6), the ascending cervical artery (C3–C4), the deep cervical artery (C3–C7), and occasionally the occipital artery (C1–C2). At C7, the supreme intercostal artery can serve as a feeder. The radiculomedullary artery from C4–C7, known as the artery of cervical enlargement, is generally the most prominent. Additionally, in the thoracic cavity, the supreme intercostal artery from the aorta (or costocervical trunk) supplies multiple intercostal levels and supports intersegmental anastomoses [25].

**Figure 2:**
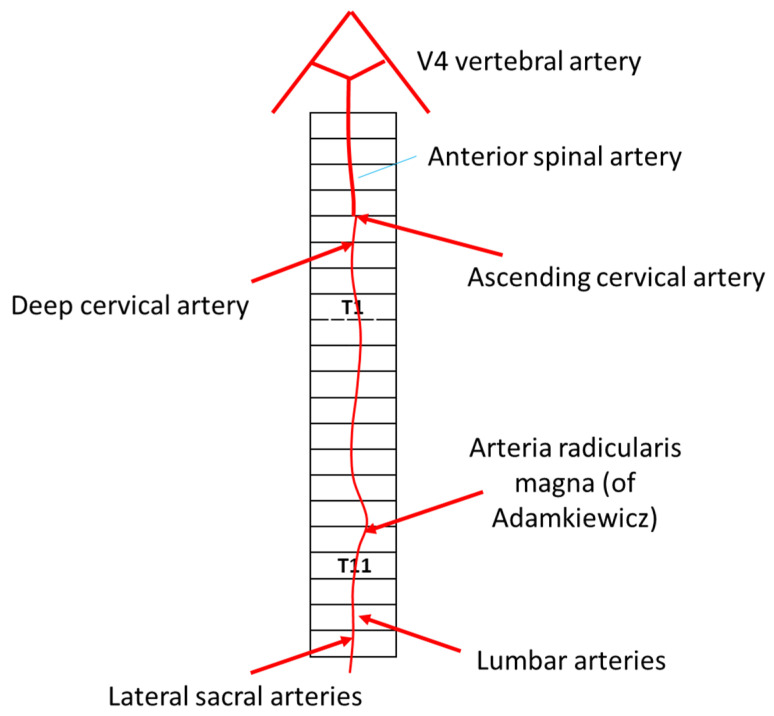
This is a schematic drawing of the arterial sources to the ASA system.

The posterior spinal arteries (PSAs), which may arise from the vertebral artery or posterior inferior cerebellar artery (PICA), run in the posterolateral sulci, supplying the dorsal columns. Circumferential branches from both the anterior and posterior spinal arteries form a pial plexus. The thoracolumbar spinal cord receives blood via 6–10 radiculomedullary feeders from the aorta and iliac arteries, with the artery of Adamkiewicz (arteria radicularis magna) being the most prominent, typically originating on the left at T8-L2 and making a characteristic hairpin turn into the ASA. The artery of lumbar enlargement, or artery of Adamkiewicz, commonly arises between T9 and T12 on the left. Figure 2 illustrates a schematic view of the arterial supply contributing to the ASA system [25].

The mid-thoracic region is more susceptible to ischemia, as the ASA may sometimes be discontinuous, whereas the cervical, lower thoracic, and lumbar regions have wide anastomoses. The radiculospinal artery (spinal segmental artery) usually terminates in reticular branches, which directly supply the spinal cord. These arteries are classified based on their supply territories: radicular (nerve root only), radiculopial (nerve root and pial plexus), and radiculomedullary (nerve root, pial plexus, and spinal cord medulla) [25,26].

**Figure 3:**
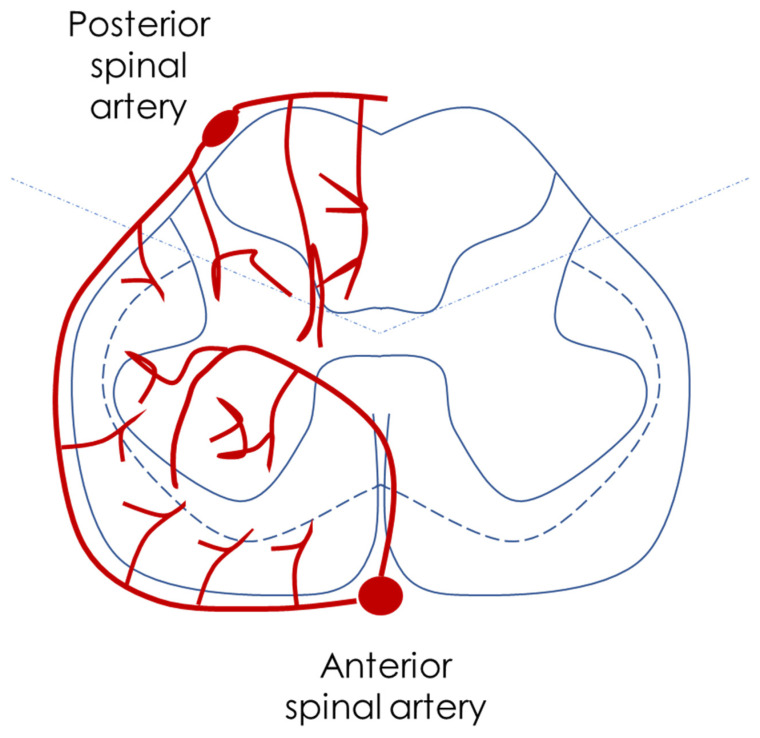
This is a schematic drawing of the arterial pattern of the spinal cord, as described in the text, highlighting the preferential supply of the anterior horns by the ASA and the watershed axial zone.

The ASA and PSA networks, particularly at the conus medullaris, are connected by a dense arterial plexus, forming an extensive pial plexus on the spinal cord’s surface. This vascular network, or vasocorona, comprises transverse and oblique branches from the ASA and PSA and supplies the spinal cord’s periphery. The intrinsic arterial system supplies the spinal cord parenchyma, and it is divided into central (centrifugal) and peripheral (centripetal or vasocorona) systems (Figure 3). The central system includes sulcal or sulco-commissural arteries from the ASA that penetrate the anterior median fissure and branch primarily within the gray matter. The peripheral system (vasocorona) consists of small perforators (rami perforantes) from the pial plexus that supply the spinal cord’s white matter. The central arteries (0.06–0.40 mm in diameter) supply the majority of the gray matter, while the peripheral arteries (0.1–0.2 mm in diameter) serve the white matter.

The ASA also supplies the ventral half of the outer white matter tracts through its contribution to the vasocorona, ultimately supporting approximately two-thirds of the spinal cord’s cross-sectional area, including the anterior commissure, anterior horns, Clarke’s nucleus, and anterior sections of the fasciculi cuneatus and gracilis, along with the corticospinal and spinothalamic tracts. The PSAs, in contrast, supply the dorsal third of the spinal cord, reaching the apex of the posterior horns. The corticospinal pathways are therefore sustained by both arterial systems.

This arrangement creates an axial watershed zone at the interface between the centripetal and centrifugal blood supplies within the spinal cord. Due to the increased vulnerability of gray matter to ischemia, early injuries in this zone often present with a distinctive “snake-eye” pattern. The unique configuration of the ASA’s ventral supply leaves the thoracic spinal cord especially susceptible to ischemic injury from occlusions or compressive pathology, as it lacks significant collateral support in this area. The extent and severity of ischemic lesions in the posterior thoracolumbar watershed region depend on multiple factors, such as the level and caliber of the major anterior and posterior radiculomedullary arteries and the specific injury mechanism (e.g., compression with partial flow reduction vs. complete occlusion, and intermittent vs. sustained flow impairment) [25,26].

Longitudinal watershed zones also exist, predominantly involving the anterior circulation, and lie between adjacent radiculomedullary territories. Their distribution is shaped by the number, size, and placement of contributing anterior radiculomedullary arteries and the type of injury. Additionally, a posterior lumbosacral watershed zone has been recently identified, with the ASA supplying the lowest portions of the PSAs via the periconal anastomotic circle, forming a longitudinal watershed zone along the dorsal surface of the conus medullaris [26].

## 4. Diagnostic Criteria

The differential diagnosis of SCI is challenging, as its acute symptoms closely resemble those of other neurological conditions, including inflammatory myelopathies, multiple sclerosis, malignancies, and infectious myelopathies. MRI is a critical tool in the diagnostic process, aiding in distinguishing SCI from these other conditions [27,28]. Recently, Zalewski et al. proposed diagnostic criteria for both spontaneous and periprocedural SCI, which incorporate clinical presentation, radiological findings, and cerebrospinal fluid (CSF) analysis [29]. These criteria provide a structured approach to improve diagnostic accuracy in cases where SCI is suspected. In the proposed series of 133 patients with a spontaneous SCI, 102 (77%) reached the nadir within 12 h, while others had a stuttering decline. MRI shows confirmatory (e.g., vertebral body infarct) and supportive findings while excluding other etiologies; CSF was usually non-inflammatory (92%). The proposed diagnostic criteria are illustrated in Table 1.

However, a previous series proposed a different definition for vascular myelopathy corresponding with SCI in three different levels of probability, as in Table 2 [30].

**Figure 4:**
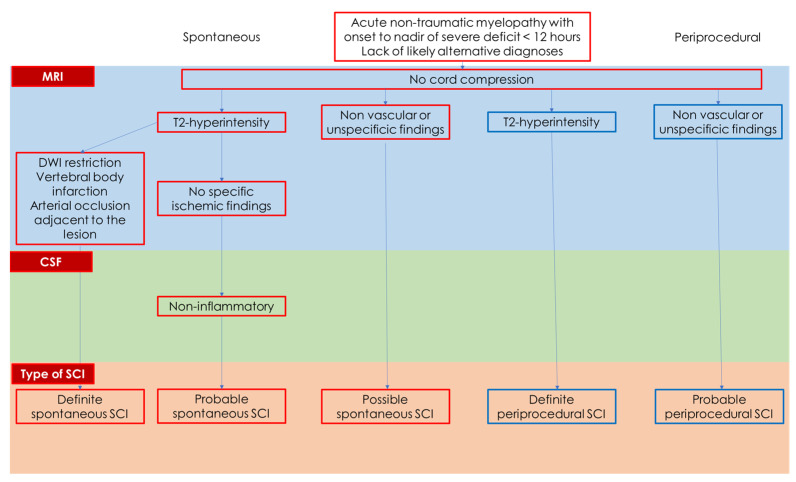
This is a schematic drawing of the issues considered in the diagnostic criteria proposed by Zalewski et al. [29].

The diagnostic criteria proposed by Zalewski et al. [29] apply to spontaneous and periprocedural SCI [29,31] and have been used to standardize a minimum diagnostic pathway, as summarized in Figure 4.

Figure 4 outlines cases where the diagnosis is not strongly supported by the available diagnostic investigations or some of these have not been performed or have been repeated shortly thereafter (e.g., spinal cord MRI with DWI sequences).

## 5. Neurological Issues

In a recent systematic review and meta-analysis [20], the most frequently reported symptoms of SCI at onset were motor deficits (92%), sensory deficits (85%), autonomic dysfunctions (76%), and pain (70%). According to the American Spinal Injury Association (ASIA) Impairment Scale score, 19% of patients had an ASIA score of A, 14% had an ASIA score of B, 36% had an ASIA score of C, and 32% had an ASIA score of D. None of the patients were neurologically unimpaired on admission. Ischemia in the ASA territory leads to neurological symptoms such as para- or tetraparesis, bladder dysfunction, bilateral sensory deficits, and impaired temperature and pain perception below the infarct level. Damage in this area also affects the anterior horns, anterolateral and crossing spinothalamic tracts, and lateral corticospinal tracts. Neurological examination may reveal variable limb temperatures due to vasoconstrictor tract involvement in the spinal cord’s lateral region. In cases of cervical infarction, ipsilateral Horner syndrome can also occur.

Infarction in the PSA territory impairs sensations like light touch, vibration, and proprioception. Unlike cerebral infarction, spinal cord ischemia is rare, with limited epidemiological data. The low incidence of medullary infarctions is likely due to the strong collateral vascular supply.

Acute pain was reported in 15% of periprocedural SCI cases, which is lower than in spontaneous cases (60–70%) [12,32]. This issue suggests that acute pain may relate more to the infarction’s underlying mechanism (e.g., dissection, fibrocartilaginous embolism) than to ischemia itself. Additionally, anesthesia and perioperative analgesia might have contributed to fewer pain reports. However, 25% of patients experienced residual neuropathic pain, emphasizing SCI’s long-term impact on quality of life [21].

In the same review [20], time to nadir (maximum severity) was reached as follows: (I) within 12 h for 81% of patients, (II) between 12 and 24 h for 11%, and (III) after 24 h for 7.7%. Another systematic review [33] showed that in 56.1% of patients, the nadir was reached within 6 h, 30.7% within 6–12 h, 5.4% within 12–72 h, and 7.8% after 72 h. A hyperacute time to nadir (<6 h) was most common (38.8%, *p* < 0.001) in thoracic lesions, while a late nadir time (12–72 h) was more frequent with cervical lesions (81.0%, *p* < 0.001). For lesions at the cervico-medullary junction, the hyperacute time to nadir was only 9.8% (*p* = 0.01).

The hyperacute presentation (within 12 h) strongly correlated with diagnostic accuracy for SCI [29,30]. In a study of 457 patients, the time of symptom onset (hyperacute < 6 h, acute 6–48 h, subacute 48 h–21 days, chronic > 21 days) was shown to have high ability to distinguish SCI from other conditions [30].

In MRI findings, 75% of patients had pathological changes on the initial scan, mostly examined 1–2 days after symptom onset, with only 10% undergoing MRI within 1 day. In those with initial negative findings, 56% were scanned within 24 h. However, follow-up MRI 1.5–42 days later showed abnormalities consistent with SCI in most cases. Diffusion-weighted imaging (DWI) was performed in 32% of cases, with 82% showing diffusion restriction. In terms of affected levels, the thoracic region was most involved (33%), followed by the cervical (24%), thoracic through lumbar (26%), cervical through thoracic (13%), isolated conus (5%), and cervical through conus (0.5%).

CSF analysis was performed in less than half of the patients; results were normal in 40%, while elevated protein levels were the most common abnormal finding (49%), with oligoclonal bands detected in 1%.

## 6. Neuroradiological Investigations

Accurate diagnosis of SCI relies on identifying and interpreting key radiographic signs. An initial emergent CTA of the chest and abdomen is recommended to exclude conditions like aortic dissection or occlusion, especially in patients with risk factors or symptoms such as chest or back pain, abnormal vital signs, or pulse discrepancies. Following this, a rapid-sequence MRI of the spine (cervical, thoracic, and/or lumbar) is suggested to rule out conditions requiring urgent intervention, such as spinal cord compression or hemorrhage. MRI should include ischemia-sensitive sequences (DWI/ADC) for early diagnosis and acute treatment. In cases with painless symptoms or unclear localization (e.g., truncal sensory level), acute head imaging may be considered to assess for atypical cerebral presentations.

SCI typically appears as T2-hyperintense signals on MRI; however, these findings may be absent within the first 24 h, due to limitations such as low spatial resolution and DWI sensitivity issues from susceptibility artifacts [34]. One study indicated DWI changes in 67% of SCI cases, though DWI sequences are not routinely applied and could benefit from advanced imaging techniques [29]. When SCI is strongly suspected, a follow-up MRI a few days later is advisable.

Characteristic MRI patterns, such as the “owl eyes” sign (bilateral T2-hyperintensity in the ventral horns on axial views) [35] and the “pencil-like” T2-hyperintensity in the anterior cord on sagittal views, are common indicators of SCI in the correct clinical context, though they are not exclusive nor required for diagnosis. Additional T2-hyperintensity patterns include anteromedial spots, anterior U/V shapes, hologrey patterns, and holocord patterns. SCI lesions tend to be longitudinally extensive (in 60% of cases) but may also be patchy or noncontiguous [29]. Lesions that span the entire cord are more suggestive of alternative diagnoses. Notably, clinical deficits in SCI may appear disproportionately severe relative to lesion size [29,31].

In the subacute phase of SCI, gadolinium enhancement often appears as a linear craniocaudal strip (in ~40% of cases), highlighting the affected vascular territory, typically the anterior or gray matter regions—an uncommon finding in inflammatory conditions [29,31]. Swelling and edema are also typical in this phase. Additional signs of SCI include adjacent vertebral body infarction or, rarely, infarction in paraspinal muscles or ribs, as well as evidence of arterial dissection or occlusion [36]. For patients with FCE, MRI may show an adjacent intervertebral disc protrusion, providing valuable diagnostic clues [29,31].

In a study by Zalewski et al. [29], who proposed diagnostic criteria for SCI, 24% of patients initially had normal MRI scans despite severe deficits when imaging was performed within 24 h of symptom onset. Follow-up imaging (median, day 4) revealed T2-hyperintense lesions in the cord, with lesion localization and imaging patterns varying along the lesion’s length. The PSA territory was involved in 45% of cases. Typical T2-hyperintense patterns in SCI included the “owl eyes” sign (65%), noncontiguous anterior “pencil-like” hyperintensity on sagittal views (40%), anterior U/V hyperintensity (25%), an anteromedial spot pattern (24%), a hologrey pattern (19%), and a holocord pattern, especially in the conus region (16%).

MRI is the primary diagnostic tool for spinal cord infarction (SCI), with hallmark findings including bilateral hyperintense lesions in the anterior horns (“owl’s eyes” sign) on axial views, pencil-like hyperintensities on sagittal sections, and hyperintensity along the anterior spinal artery. In a pooled analysis of 371 patients, MRI revealed positive findings in 279 cases, with most scans performed within 48 h and only 10% within the first 24 h [20]. However, early-stage MRI sensitivity is low; up to 50% of T2-weighted images may not show lesions within the first 24 h [37,38,39]. Among patients with initially normal MRIs, follow-up imaging (performed 1.5 to 42 days later) showed SCI-consistent findings in 92% of cases [20]. Despite low early sensitivity, MRI remains crucial in differentiating SCI from other conditions requiring different acute interventions.

DWI could enhance early ischemic lesion detection in SCI, similar to its use in stroke. However, spinal column heterogeneity, blood supply variability, and CSF pulsations complicate DWI for spinal imaging [34,40,41]. Limited data exist on the role of DWI in SCI, with only 87 patients undergoing DWI in a recent systematic review [20]. Nonetheless, DWI might improve diagnostic accuracy and help in ruling out alternative diagnoses. In a recent systematic review [33], T2 DWI sequences were positive in only 22.3% of cases initially, with the characteristic T2 “owl’s eyes” hyperintensity observed in 48.2% of cases. The proportion of initial T2 sequence positivity with diffusion restriction was significantly higher at the cervical (35.9%), cervico-thoracic (32.3%), and thoracic (22.6%) levels. The “owl’s eyes” sign was most frequently reported at the cervical (39.6%) and thoracic levels (22.9%).

MRI is the preferred imaging technique for SCI, though its sensitivity in the initial stages is low. Abnormalities often do not appear on standard T1- and T2-weighted images for several hours or even days, and follow-up scans typically reveal signal changes. The development of DWI has improved MRI sensitivity for detecting arterial ischemic damage.

The primary neuroimaging characteristics of SCI include the following conditions:

- Predominant involvement of the thoracic cord and conus, typically within the ASA territory;
- Initial MRI may appear normal;
- Restricted diffusion, with a high signal on DWI and low signal on apparent diffusion coefficient (ADC) images, is similar to arterial infarcts in the brain;
- ASA infarcts on T2-weighted images commonly show central gray matter involvement, with an H- or butterfly-shaped appearance, or the “owl’s eyes” or “snake-eyes” sign;
- Central gray matter involvement is characteristic but not definitive for diagnosis;
- PSA infarcts, though less common, affect the posterolateral third of the cord, including the dorsal columns and posterior horns;
- In the hyperacute and acute phases, parenchymal T2-hyperintensity appears non-expansile, thin, and pencil-like on sagittal images;
- In the later acute and early subacute phases, spinal cord enlargement may occur;
- Parenchymal enhancement, often linear and involving the anterior gray matter, may appear in the acute–subacute phase;
- Hemorrhagic transformation is rare;
- Vertebral body infarcts may also be observed.

Finally, the diagnosis of SCI by imaging is based on a combination of clinical and radiological elements: topology of the lesions relative to vascular anatomy, diffusion abnormalities, and progression observed on images. The most frequently damaged vascular territory (90%) is that of the ASA, predominantly in the thoracolumbar area. There is central damage, predominantly affecting gray matter, with a hypersignal on T2-WI that may appear as late as 48 h after onset. A typical pencil-like appearance is observed on sagittal sequences in almost 100% of MRI-positive cases. An enlargement of the spinal cord may be observed in about 40% of cases. On axial slices, abnormalities are observed mainly in the anterior two-thirds of the spinal cord and gray matter, corresponding to the intrinsic spinal cord microvascularization of the ASA. Damage exclusively affecting the anterior horns, as observed in junctional infarction, results in an ‘‘owl’s eyes’’ appearance. Such damage occurs following hypovolemia or hypoperfusion. This appearance is observed in only 7% to 33% of cases, depending on the study and etiology considered. The abnormalities are usually bilateral and may vary with individual anatomy and the artery concerned, but they may also be unilateral. The lesions appear usually as a hyposignal on T1-WI, although a few cases of a peripheral T1-WI hypersignal due to hemorrhagic transformation have been reported (11%). On DWI, a hypersignal with a restricted ADC can confirm the diagnosis and distinguish spinal cord infarcts from other lesions. The mean ADC of an ischemic lesion is lower than that of the spinal cord of control subjects, whereas the ADC of inflammatory myelitis is higher. Abnormalities on DWI have been reported from the third hour after the onset of signs, with a normalization of the ADC from the seventh day onwards, but the kinetics of the appearance of lesions has not yet been clearly established. A diffusion hypersignal coexists with a T2-WI hypersignal in most cases, probably due to the time between the appearance of signs and the performance of MRI. Contrast enhancement in the ischemic zone of the cord, due to rupture of the blood–cord barrier, may occur after several days, but is not observed in the initial phase. Contrast enhancement of the anterior roots, particularly the cauda equina, has been reported between the third and fourth days.

A summary of the main neuroimaging features of SCI in the acute, subacute, and chronic phases is illustrated in Table 3.

While the presumptive diagnosis is based on the typical presentation and clinical findings, T2-weighted and DWI-MRI were the most useful diagnostic tools in establishing a definitive diagnosis [42]. Some differences have been reported between spontaneous and periprocedural SCI regarding the extent of spinal cord lesions. The main features of periprocedural SCI were highlighted by Zalewski et al. in a case series [31], confirming MRI patterns that aid in the diagnosis, especially for ischemia affecting the anterior two-thirds of the spinal cord. In periprocedural SCI as well, typical features include the “owl eyes” sign [43] and the “pencil sign” [44], both of which are found in around 70% of cases. A “hologrey” T2-hyperintensity pattern on axial images is also common, indicating the susceptibility of gray matter to ischemic injury. Lesions often are irregular, are non-continuous, or display distinct anterior T2-hyperintensity shapes, such as anteromedial spots or “U” and “V” shapes [12,35]. They tend to span multiple vertebral segments (three or more), from the thoracic region down to the conus, and are frequently accompanied by cord swelling or edema. It is noteworthy that acute MRI findings may initially appear normal in SCI cases, which should heighten suspicion when clinical signs suggest SCI [45]. This radiographic delay resembles patterns seen in acute cerebral stroke, where about 7% of cases in posterior circulation strokes initially appear normal on MRI [45,46]. DWI and ADC sequences can reveal restricted diffusion in approximately half of SCI cases, offering valuable diagnostic support for ischemic myelopathy, despite imaging limitations with the spinal cord.

**Figure 5:**
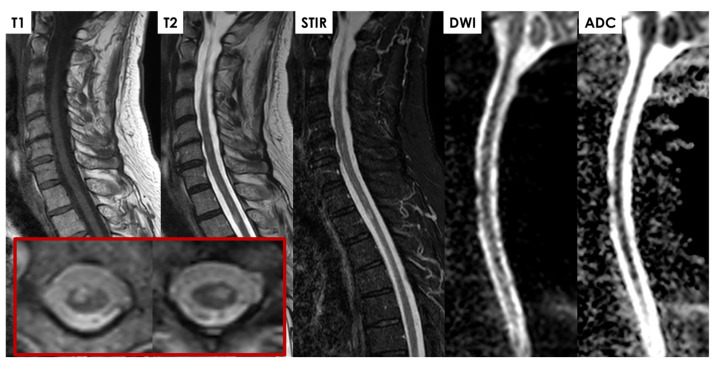
A 78-year-old patient with multiple vascular risk factors underwent vascular surgery for a thoraco-abdominal aortic aneurysm, with complications during awakening due to inability to extubate, which was performed three days later. After extubation and awakening, hyposthenia was noted in all four limbs, asymmetric (but predominantly affecting the lower limbs), with areflexia in the lower limbs and hypo-responsive proprioceptive reflexes in the upper limbs. The patient was catheterized preoperatively, with sphincter function not fully assessable. Sensory levels were unclear. An MRI study was performed 5 days post-surgery under the best possible conditions (hemodynamically stable patient but in fragile balance, on high-flow therapy, GFR < 30 mL/min, etc.). Multifocal intramedullary lesions in the high cervical–thoracic segment (at least 4) were hyperintense on T2 with different lateralization on axial scans (red lined figures) and involvement of the anterior horns (the left axial image corresponds to the highest lesion at C6-C7, and the right one to the lesion at the D3 level). DWI/ADC findings are consistent with timing (5 days post-surgery). Contrast administration was not performed due to renal failure, and the non-contrast study was already diagnostic given the clinical context.

**Figure 6:**
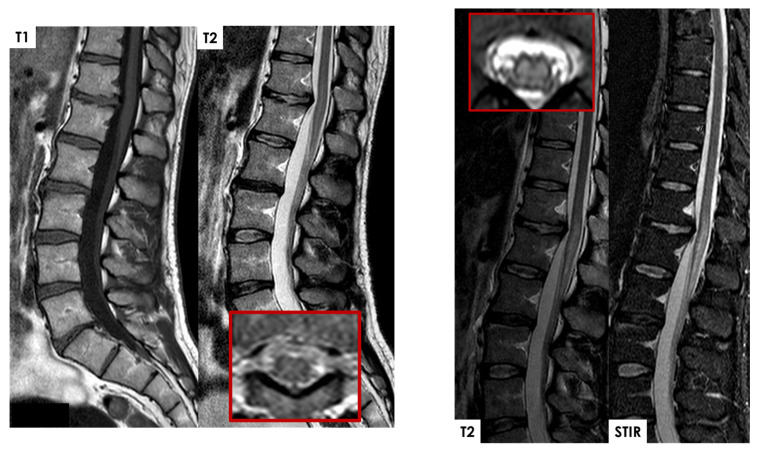
A 38-year-old patient in good health with moderate habitual physical activity. While performing gymnastics at home, the patient experienced the sudden onset of lumbar sensitive deficit, with rapid radiation to both lower limbs, associated with painful symptoms (described as cramp-like) in the same areas. This was followed by hyposthenia in both lower limbs, progressively worsening to the point of being unable to maintain an upright posture. Upon arrival at the emergency department, urinary retention was documented, and a catheter was placed. The patient underwent a CT angiography of the aorta, with a report showing no pathological findings, as well as a spinal MRI that was reported as normal (with a minimal osteophytic protrusion at the D10-D11 level extending posteriorly) (red lined figure). The following day, the patient had a repeat spinal MRI, which showed an intramedullary lesion hyperintense on T2W sequences at the D12-L1 level, with almost full axial extension (red lined figure), without bone alterations suggestive of trauma (DWI not available). The initial diagnosis was post-traumatic injury, followed by a suspicion of myelitis (subsequent diagnostic workup was negative for immune-mediated diseases). Outcome: ASIA D paraplegia from cord contusion at D12-L1 with motor level L4 on the right and L5 on the left, and sensory level L3 bilaterally. Neurogenic bowel and bladder. Most appropriate diagnostic hypothesis based on clinical and neuroradiological data: spinal ischemia. Spinal angiographic study (DSA) was performed after referral for a second opinion 8 months after the event: negative. The images on the right side of the slide are from the MRI performed 24 h after symptom onset.

**Figure 7:**
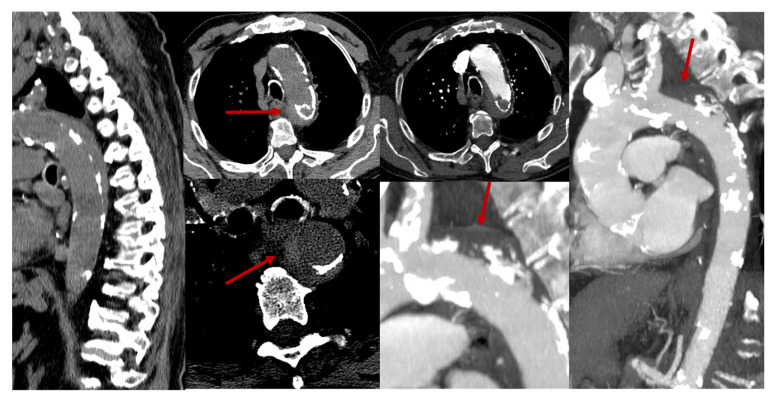
An 80-year-old patient reported severe acute back pain, followed by immediate onset of paraplegia with sensory level at D3-D4. An emergent CT angiography of the thoracic and abdominal aorta diagnosed an aortic dissection. Even in the pre-contrast sagittal reconstruction scan, an eccentric, crescent-shaped, isodense thickening was clearly visible in the wall of the thoracic aorta, just after the origin of the left subclavian artery, delineated by coarse calcifications on the intimal side of the aortic wall, highly suggestive of a mural hematoma. In the axial scans, a slightly hyperdense component was also outlined, possibly indicating ongoing bleeding. The post-contrast study confirmed the finding, with better delineation of the endoluminal dissection of severe and complicated aortic atheromatosis, where the fracture of the fibrous cap (in the segment without calcifications) could have created the entry point for the formation of the mural hematoma (red arrows).

**Figure 8:**
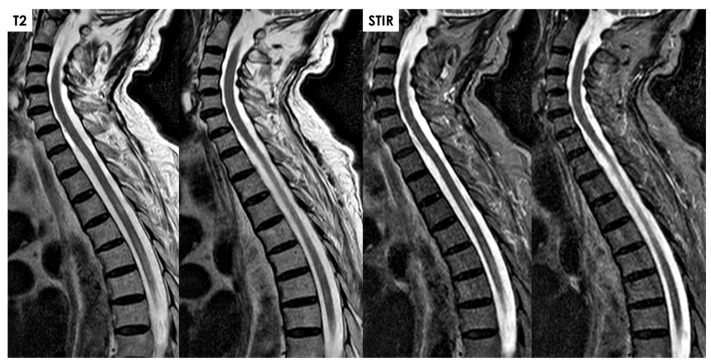
Same patient as in Figure 6. On the MRI performed three days after the onset, multifocal intramedullary lesions are evident, with extravasation at the level of the dorsal spinal cord. The first lesion spans from D3 to D4 for nearly two vertebral bodies, and the second spans D5-D6-D7 for at least three vertebral bodies, showing hyperintensity in T2 and STIR sequences, consistent with ischemic lesions. Axial scans and DWI are compromised by motion and respiratory artifacts.

Subacutely, gadolinium enhancement often highlights ischemic changes in the ventral roots of the cauda equina, which may initially suggest inflammation, infection, or malignancy but instead likely reflects compromised blood–nerve barriers in ischemic nerve roots [43,47,48,49,50,51,52]. Gadolinium also frequently enhances the infarcted spinal cord itself in subacute cases, pinpointing areas of significant ischemia against a background of edema, often showing anterior horn cell enhancement. In some cases, a T2-hyperintense signal along the anterior spinal artery near the SCI site can suggest thrombotic activity or reduced blood flow [47,53]. Rarely, adjacent vertebral body infarction further supports a diagnosis of SCI by clarifying the extent and nature of the ischemic event. Some examples of periprocedural and spontaneous SCI are illustrated in Figure 5, Figure 6, Figure 7 and Figure 8.

In case series of SCI, the high prevalence of older patients with vascular risk factors suggests that traditional stroke mechanisms, such as atherothrombosis, may significantly contribute to SCI, particularly by affecting larger proximal arteries rather than smaller spinal arteries [54,55]. Cervical artery dissections, however, are often missed in standard angiographic imaging, but T1-weighted fat suppression imaging can help visualize a vessel wall hematoma, confirming dissection [56]. Additionally, identifying nearby disc protrusions could suggest FCE as a cause of SCI, though degenerative disc disease is also common in the general population [57].

In the study by Zalewski et al. [29], vascular imaging—using CT angiography, MR angiography, or digital subtraction angiography (DSA)—was performed in 63% of patients, with 20% revealing specific vascular abnormalities, including aortic dissection, vertebral artery dissection, anterior spinal artery occlusion, aortoiliac occlusion, and artery of Adamkiewicz occlusion. Among patients with suspected FCE, 53% had intervertebral disc extrusions adjacent to the lesion. Given the challenges of detecting dissections, a specific imaging protocol (e.g., cervical MRI with T1-weighted fat saturation) is recommended. Dissections and mechanical issues affecting smaller arteries may underlie some idiopathic cases. Although spinal MRA has limited utility in SCI diagnosis, it can be useful if a rare acute spinal dural arteriovenous fistula (SDAVF) or arteriovenous malformation (AVM) is suspected. In rare cases, long occlusions in the anterior spinal artery or large arteries are found. However, DSA is generally reserved for cases with high suspicion of an alternative diagnosis, such as SDAVF or a rare etiology like vasculitis, due to its cost and associated risks [58,59].

The main neuroradiological features of SCI, which have been exposed both in the acute phase and in the evolution of the disease, are of particular value to differentiate SCI from other causes of spinal cord injury, for example inflammatory causes. In fact, an adequate knowledge of the vascularization and vascular territories of the spinal cord, including the microvascularization and the functional organization of the watershed zones, the use of DWI-MRI, and, when available, the contrast enhancement pattern, allows one to make a diagnosis of SCI versus other myelopathies, in particular in patients with acute onset of symptoms and onset to nadir time < 12 h.

## 7. Etiologies

Two main etiologies of SCI have been recognized: periprocedural and spontaneous SCI. Periprocedural SCI often occurs as a complication of vascular surgery, affecting blood supply to the spinal cord. The remaining SCI is spontaneous, with pathophysiology resembling that of cerebral strokes.

The causes and implications of SCI vary between children and adults. In children, SCI is often due to cardiovascular abnormalities and trauma [59,60]. In adults, aortic disease is the primary cause, often linked to conditions like atherosclerosis, aortic surgery, and thoracic aortic aneurysms [14,21,61]. A history of vascular disease increases the risk of SCI, with factors such as hypertension, smoking, hyperlipidemia, and diabetes mellitus being the main contributors [29,61]. Other triggers of SCI include adjacent spinal degenerative disease, spinal or epidural anesthesia, FCE associated with disc herniation, vertebral artery dissection, sympathectomy, systemic hypotension, cardiac embolism, coagulopathies, vasculitic disorders, surfer’s myelopathy, and decompression sickness [62,63,64,65,66,67]. Additional risk factors include cocaine use, physical maneuvers like heavy lifting and the Valsalva maneuver (often linked to early onset), nucleus pulposus herniation, and chiropractic manipulation [17,68]. Septic thrombophlebitis can also lead to spinal venous occlusion, causing ischemic infarction [69].

Surgery to repair thoracic and thoraco-abdominal aortic aneurysms is the most common cause of spinal cord infarction [15,44,70]. Rates of SCI following these surgeries can be as high as 29%, though they are more commonly reported in a range between 10 and 11% [71]. Both open surgery and endovascular repair carry risks of SCI, with some evidence suggesting lower risks for endovascular approaches, though this may be influenced by patient selection bias [72,73,74,75].

SCI may present immediately after surgery or following a period of normal neurologic function, with delayed cases occurring up to 27 days after surgery [76,77]. Contributing factors include systemic hypotension, aortic cross-clamping (leading to reduced arterial perfusion and increased spinal canal pressure), and occlusion of critical blood vessels, such as the artery of Adamkiewicz or intercostal arteries, due to ligation, resection, or embolization. Systemic hypotension is often associated with delayed ischemic onset [76,77].

Risk factors for SCI after aneurysm repair include advanced age, aortic rupture, cerebrovascular disease history, prior aortic surgery, extensive aortic disease (e.g., Crawford II/III repairs), postoperative bleeding, prolonged cross-clamping, intraoperative hypotension, intercostal vessel sacrifice, impaired kidney function, and atrial fibrillation [77,78,79].

Preventive strategies to lower the risk of SCI include lumbar drainage, reimplantation of intercostal arteries, intraoperative neurophysiologic monitoring, epidural cooling, distal aortic perfusion, and arterial blood pressure management. While these interventions show promising results, they have not yet been evaluated in randomized or rigorously controlled studies [77,78,79,80].

Acute dissection of the descending aorta is often catastrophic, with a high mortality rate ranging from 10% to 50%. Survivors frequently face complications due to the acute occlusion of branch vessels, including the celiac, superior mesenteric, and renal arteries, as well as radicular arteries supplying the spinal cord. SCI occurs in approximately 4% of aortic dissections, though it is rare for SCI to be the initial symptom of aortic dissection, though documented in several cases [81,82,83,84,85]. SCI associated with aortic dissection generally affects the mid- to lower thoracic levels [86]. Severe “tearing” pain and abnormal distal pulses are typical indicators, though 5% to 15% of acute aortic dissections are painless, requiring high clinical suspicion for accurate diagnosis. Risk factors for aortic dissection include chronic hypertension, underlying atherosclerotic disease, and Marfan syndrome [87,88].

Peripheral venoarterial extracorporeal membrane oxygenation (V-A ECMO) is a critical life support technique used in cases of severe circulatory or cardiac failure. In V-A ECMO, blood is drained from a central vein, oxygenated externally, and reinfused into an artery. This process involves large vessel cannulation, cardiopulmonary bypass, and anticoagulation. Complications of V-A ECMO can include SCI, possibly due to retrograde blood flow in the aorta, leading to stasis in the aortic root or left ventricle, or thromboembolism caused by occlusion of spinal radicular arteries from iliac arteries and turbulent aortic flow [89].

SCI can also complicate traumatic aortic rupture and other aortic conditions, such as aneurysms, thrombosis, and, less commonly, coarctation of the aorta or its surgical correction [38,44,90,91]. Additionally, aortography has been linked to SCI in some cases [92].

Various other surgeries have been associated with SCI, with spine surgery being the most common. Other procedures include bowel resection, hepatectomy, cesarean section, and hip and prostate surgeries, as well as endovascular interventions like percutaneous coronary and neurointerventional procedures [93,94,95]. In some cases, the injury may stem from damage to a radicular feeding artery, epidural anesthesia-induced arterial injury or vasospasm, or intraoperative hypotension. Patients with underlying aortic disease or previous aortic surgeries may be more susceptible to perioperative SCI [96,97,98,99,100].

FCE is a rare but serious cause of SCI originating from herniated intervertebral discs. FCE typically affects a wide age range (7–78 years), with most cases involving the cervical cord [101,102,103,104,105]. Symptoms are often preceded by minor neck trauma or heavy lifting, with neck pain occurring minutes to days before neurological deficits. MRI may reveal a collapsed intervertebral disc at the affected level, and autopsy findings sometimes show fibrocartilaginous material in blocked spinal arteries, although hyperextension and vascular compression may also play a role [106,107,108].

In addition, the spinal cord is particularly vulnerable to ischemic injury during systemic hypotension, as can be seen in cardiopulmonary arrest or major blood loss. Autopsy studies show ischemic myelopathy in nearly half of adults who die following cardiac arrest or severe hypotensive episodes, primarily affecting the lumbosacral region [109,110]. This issue is also noted in neonates, especially those born prematurely, who experience perinatal hypotension. Among survivors, hypoxic–ischemic encephalopathy may mask ischemic myelopathy, though spinal cord infarction remains a significant complication in some cases [111].

Finally, in case series, 44% to 74% of patients with SCI lack an identifiable cause. Many of these patients have atherosclerotic risk factors, suggesting that atherothrombotic disease may contribute to some cases, though specific pathology is rarely described. Additionally, some patients present without any identifiable cause or vascular risk factors [12].

Other documented causes of SCI include (I) vasculitis due to infections (bacterial or syphilitic), systemic lupus erythematosus, polyarteritis nodosa, and giant cell arteritis; (II) vertebral artery atheroma and dissection, associated with cervical cord infarction; (III) inherited and acquired hypercoagulable conditions, like prothrombin gene mutations and sickle cell disease; (IV) cervical spondylosis, which may lead to infarction via dissection or compression of radicular arteries; (V) transforaminal epidural glucocorticoid injections, which can cause arterial spasm, dissection, or embolism due to intraarterial injection; (VI) umbilical artery catheters in newborns, potentially obstructing the artery of Adamkiewicz; (VII) cocaine-related arteriopathy, implicated in a few cases of spinal cord infarction; (VIII) cardiogenic emboli, originating from artificial valves or vegetations, which can lead to SCI; (IX) bronchial artery embolization used for hemoptysis treatment, which may cause embolic SCI; and (X) decompression sickness myelopathy, potentially vascular in origin [112,113,114,115,116,117,118,119,120,121,122,123,124,125,126,127,128,129,130,131,132,133,134,135,136,137].

These diverse etiologies highlight the complexity and multifactorial nature of SCI.

## 8. Gray Areas

The proposed definitions and diagnostic criteria for SCI aim to facilitate diagnosis of this often underrecognized condition, frequently misdiagnosed as transverse myelitis [30,138]. Three main criteria guide diagnosis: (1) the rapid progression of severe symptoms to their peak within 12 h; (2) MR-based neuroimaging that excludes compression while supporting SCI with specific imaging features; and (3) CSF findings that are non-inflammatory. The critical factor is the rapid symptom onset within 12 h, which is an uncommon presentation for other myelopathies and thus helps differentiate SCI. However, some SCI cases, including those confirmed by autopsy, have shown prolonged symptom progression. This slower evolution may be due to the spinal cord’s collateral vascular network, which could temporarily sustain tissue viability before final infarction. While the ASA supplies most of the spinal cord, a notable portion of SCI cases involve the PSA territory. The ASA, supplying the anterior two-thirds of the spinal cord, contrasts with the dual PSA, which supplies the posterior third. Additionally, the spinal cord has an extensive anastomotic network of penetrating vessels along its surface, particularly at cervical levels, where it is richly vascularized. This complex vascular network could account for intermediate zones that can variably involve both anterior and posterior regions of the spinal cord [139,140]. Pain is commonly reported at SCI onset (72%) and may result from spinothalamic tract activation or other contributory factors, such as fibrocartilaginous embolism or artery dissection [41,141,142].

Certain MRI findings can confirm SCI, although some signs are often absent, and essential sequences may not be performed. DWI can support SCI diagnosis in acute cases of myelopathy, despite its limited sensitivity due to the spinal cord’s spatial resolution and susceptibility artifacts. Classic MRI indicators, like the “owl eyes” or pencil-like T2W hyperintensity, can suggest SCI, but are not specific or necessary for diagnosis. SCI can initially appear normal on MRI (24% of cases in Zalewski et al. [29]), so repeat imaging after a few days is recommended if clinical suspicion remains. Although gadolinium enhancement is often linked with transverse myelitis or similar conditions, 43% of periprocedural and 39% of spontaneous SCI cases show enhancement. Typical SCI gadolinium patterns include a craniocaudal line of enhancement, generally along the gray matter, which is uncommon in inflammatory conditions and can help pinpoint the affected arterial area. In uncertain cases, chronic focal cystic myelomalacia visible on follow-up imaging suggests vascular damage over myelitis [102,143]. Further, a key diagnostic challenge is the accuracy of available tools. A systematic review highlighted moderate accuracy of T2/DWI in detecting T2-hyperintensity (T2HSI) in hyperacute cases (symptoms < 6 h). ROC analysis showed an AUC of 0.835 with a cutoff of 0.5, indicating moderate accuracy for T2/DWI at the hyperacute phase [33]. MRI remains the preferred diagnostic tool for SCI, showing pencil-like hyperintensities on T2-weighted images and an “owl eye” pattern in axial images when gray matter is affected. T1-weighted imaging may reveal hyperintensity from hemorrhagic transformation or infarction in adjacent vertebral bodies in some cases [144]. Gadolinium enhancement is common, although some patients show no MRI changes, especially if imaging occurs shortly after symptom onset. In such cases, DWI sequences are more sensitive [29]. However, DWI quality may be limited in spinal cord imaging due to flow artifacts, proximity to bone, and spinal cord anatomy. DWI sensitivity at hyperacute time points (<6 h) varies with lesion location, being highest in thoracic lesions (38.8%), and increases at later stages (12–72 h), with 81% sensitivity [33]. This suggests that T2/DWI has moderate sensitivity for T2HSI in hyperacute SCI cases.

## 9. Acute Treatment

Acute treatment of spinal infarction with fibrinolysis remains a controversial and rarely implemented approach due to the limited evidence and the complex vascular anatomy of the spinal cord. SCI, though uncommon, can result from arterial occlusion or embolism, leading to ischemia and subsequent neurological deficits. In selected cases, fibrinolytic therapy, such as intravenous recombinant tissue plasminogen activator (rtPA), may be considered, if initiated within a narrow therapeutic window, typically under 4.5 h from symptom onset, and only after meticulous exclusion of contraindications like spinal hemorrhage, underlying vascular malformations, or coagulopathies. Imaging techniques, such as spinal MRI with DWI, are critical for rapid diagnosis and guiding treatment. However, the use of fibrinolysis is often constrained by the risk of hemorrhagic complications and the lack of robust clinical trials supporting its safety and efficacy in this setting [145,146]. Consequently, the decision to perform thrombolysis must be individualized, balancing potential benefits against significant risks, and preferably conducted in specialized centers with expertise in neurovascular emergencies.

## 10. Conclusions

SCI is a rare vascular disease and a challenging diagnosis, requiring an appropriate clinical context to raise clinical suspicion, neuroradiological issues to be interpreted within this context (even with negative MRI findings), and a comprehensive diagnostic pathway that has not yet been stated. The proposed diagnostic criteria of SCI may help to consider the diagnosis in atypical cases too, but they have not been widely applied until now. However, in all considered series, the main issue supporting the diagnosis is clinical information, i.e., the onset to nadir time of severe involvement < 12 h. If even this single element were widely known and applied in clinical practice, it is likely that misdiagnosis of SCI due to other causes (often inflammatory) would be less common and that many cases would not be misclassified, thus contributing to a better definition of the disease characteristics and to the selection of patients for further treatment.

## Figures and Tables

**Table 1:** Diagnostic criteria of SCI, as proposed by Zalewski et al. [29].

Criteria		
1	Acute non-traumatic myelopathy (no preceding progressive myelopathy) Onset to nadir of severe deficits ≤ 12 h If stuttering course is >12 h, severe deficits rapidly develop in 12 h or less	
2	MRI A. No spinal cord compression B. Supportive: Intramedullary T2-hyperintense spinal cord lesion C. Specific (1 of): Diffusion-weighted imaging/apparent diffusion coefficient restriction, associated vertebral body infarction, arterial dissection/occlusion adjacent to lesion	
3	Non-inflammatory CSF (normal cell count, IgG index, and no oligoclonal bands)	
4	Alternative diagnosis is not likely	
Probability Level		
Definite spontaneous SCI		1, 2A, 2B, 2C, 4
Probable spontaneous SCI		1, 2A, 2B, 3, 4
Possible spontaneous SCI		1, 4
Definite periprocedural SCI		1, 2A, 2B, 4
Probable periprocedural SCI		1, 4

**Table 2:** Alternative diagnostic clues for vascular myelopathy of arterial origin with probability degrees [30].

Probability of the Diagnosis of Vascular Myelopathy		
Definite	Probable	Possible
MRI hyperintense lesion in a defined vascular territory or watershed area on T2- weighted images	MRI hyperintense lesion in a defined vascular territory or watershed area on T2-weighted images	MRI hyperintense lesion in a defined vascular territory or watershed area on T2-weighted images
Vascular abnormality demonstrated on spinal angiogram explanatory of the clinical presentation	Spinal angiogram negative or not available	Spinal angiogram and DWI negative or not available
Exclusion of other etiologies	Positive DWI or known stroke risk factors or mechanism explanatory of the clinical presentation (i.e., severe hypotension, hypercoagulable state)	No identifiable risk factor or mechanism
	Exclusion of other etiologies	Exclusion of other etiologies

**Table 3:** MRI features of SCI.

Acute Phase
- DWI/ADC should be performed, but the sensitivity is incomplete (50–70%) and sometimes takes days to evolve. - In the initial hours of symptoms, imaging is likely normal or equivocal. - Early T2-hyperintense signal typical of spinal cord infarction may be seen. - Significant edema/swelling or enhancement is unusual acutely. - Adjacent dissection/occlusion or concurrent cerebral infarction may also be identified.
Subacute Phase
- A variety of axial T2-hyperintensity patterns can be seen in the day(s) after spinal cord infarction. - The lower thoracic cord into the conus frequently demonstrates larger cross-sectional lesion areas that do not respect classic vascular territories. - When present (in approximately 40% of cases), gadolinium enhancement in spinal cord infarction is usually a linear craniocaudal strip; resolution of enhancement is typically seen within months. - Specific findings confirming spinal cord infarction include diffusion restriction, vertebral body infarction, and adjacent arterial dissection/occlusion. - Well-defined DWI hyperintensity should be confirmed on ADC. - MRA of the spinal canal is currently of limited value in spinal cord infarction.
Chronic Phase
- Several weeks to months after a spinal cord infarction, differentiating imaging findings from other causes of myelopathy becomes more difficult. - Although, at times, classic findings of myelomalacia (very bright focal T2-hyperintensity with T1 hypointensity and volume loss), cord atrophy, or residual T2-hyperintensity respecting a vascular territory can be seen, many cases are more difficult to distinguish.

## Data Availability

Not applicable.
